# Supplementary material for: A fault-intrusion-tolerant system and deadline-aware algorithm for scheduling scientific workflow in the cloud
Source: PeerJ Comput Sci. 2021 Nov 2;7:e747. doi: 10.7717/peerj-cs.747 (PMC8576554; doi:10.7717/peerj-cs.747)
Supplement: Supplemental Information 6 [file peerj-cs-07-747-s006.zip › Myproject1.1/docs/org/workflowsim/WorkflowScheduler.html]

WorkflowScheduler


JavaScript is disabled on your browser.


- Overview
- Package
- Class
- Use
- Tree
- Deprecated
- Index
- Help

- Prev Class
- Next Class

- Frames
- No Frames

- All Classes

- Summary:
- Nested |
- Field |
- Constr |
- Method

- Detail:
- Field |
- Constr |
- Method


org.workflowsim

## Class WorkflowScheduler

- java.lang.Object
- - org.cloudbus.cloudsim.core.SimEntity
  - - org.cloudbus.cloudsim.DatacenterBroker
    - - org.workflowsim.WorkflowScheduler

- All Implemented Interfaces:
  :   java.lang.Cloneable

  ---

    

  ```
  public class WorkflowScheduler
  extends DatacenterBroker
  ```

  WorkflowScheduler represents a scheduler acting on behalf of a user. It hides
  VM management, as vm creation, sumbission of jobs to this VMs and destruction
  of VMs.

  Since:
  :   WorkflowSim Toolkit 1.0

- - ### Field Summary

    - ### Fields inherited from class org.cloudbus.cloudsim.DatacenterBroker

      `cloudletList, cloudletReceivedList, cloudletsSubmitted, cloudletSubmittedList, datacenterCharacteristicsList, datacenterIdsList, datacenterRequestedIdsList, vmList, vmsAcks, vmsCreatedList, vmsDestroyed, vmsRequested, vmsToDatacentersMap`
    - ### Fields inherited from class org.cloudbus.cloudsim.core.SimEntity

      `FINISHED, HOLDING, RUNNABLE, WAITING`
  - ### Constructor Summary

    Constructors

    Constructor and Description

    `WorkflowScheduler(java.lang.String name)`

    Created a new WorkflowScheduler object.
  - ### Method Summary

    Methods

    | Modifier and Type | Method and Description |
    | --- | --- |
    | `void` | `bindSchedulerDatacenter(int datacenterId)` Binds this scheduler to a datacenter |
    | `protected void` | `processCloudletCheck(SimEvent ev)` process cloudlet (job) check (not supported yet) |
    | `protected void` | `processCloudletReturn(SimEvent ev)` Process a cloudlet (job) return event. |
    | `protected void` | `processCloudletSubmit(SimEvent ev)` Submits cloudlet (job) list |
    | `protected void` | `processCloudletUpdate(SimEvent ev)` Update a cloudlet (job) |
    | `void` | `processEvent(SimEvent ev)` Process an event |
    | `protected void` | `processResourceCharacteristicsRequest(SimEvent ev)` Process a request for the characteristics of a PowerDatacenter. |
    | `protected void` | `processVmCreate(SimEvent ev)` Process the ack received due to a request for VM creation. |
    | `void` | `setWorkflowEngineId(int workflowEngineId)` Sets the workflow engine id |
    | `void` | `shutdownEntity()` Terminate this entity (WorkflowScheduler) |
    | `void` | `startEntity()` Start this entity (WorkflowScheduler) |
    | `protected void` | `submitCloudlets()` Submit cloudlets (jobs) to the created VMs. |

    - ### Methods inherited from class org.cloudbus.cloudsim.DatacenterBroker

      `bindCloudletToVm, clearDatacenters, createVmsInDatacenter, finishExecution, getCloudletList, getCloudletReceivedList, getCloudletSubmittedList, getDatacenterCharacteristicsList, getDatacenterIdsList, getDatacenterRequestedIdsList, getVmList, getVmsAcks, getVmsCreatedList, getVmsDestroyed, getVmsRequested, getVmsToDatacentersMap, incrementVmsAcks, processOtherEvent, processResourceCharacteristics, setCloudletList, setCloudletReceivedList, setCloudletSubmittedList, setDatacenterCharacteristicsList, setDatacenterIdsList, setDatacenterRequestedIdsList, setVmList, setVmsAcks, setVmsCreatedList, setVmsDestroyed, setVmsRequested, setVmsToDatacentersMap, submitCloudletList, submitVmList`
    - ### Methods inherited from class org.cloudbus.cloudsim.core.SimEntity

      `cancelEvent, clone, getEventBuffer, getId, getName, getNextEvent, getNextEvent, getState, numEventsWaiting, numEventsWaiting, pause, run, schedule, schedule, schedule, schedule, scheduleFirst, scheduleFirst, scheduleFirst, scheduleFirst, scheduleFirstNow, scheduleFirstNow, scheduleFirstNow, scheduleFirstNow, scheduleNow, scheduleNow, scheduleNow, scheduleNow, selectEvent, send, send, send, send, sendNow, sendNow, sendNow, sendNow, setEventBuffer, setId, setState, waitForEvent`
    - ### Methods inherited from class java.lang.Object

      `equals, finalize, getClass, hashCode, notify, notifyAll, toString, wait, wait, wait`

- - ### Constructor Detail


    - #### WorkflowScheduler

      ```
      public WorkflowScheduler(java.lang.String name)
                        throws java.lang.Exception
      ```

      Created a new WorkflowScheduler object.

      Parameters:
      :   `name` - name to be associated with this entity (as required by
          Sim\_entity class from simjava package)

      Throws:
      :   `java.lang.Exception` - the exception
  - ### Method Detail


    - #### bindSchedulerDatacenter

      ```
      public void bindSchedulerDatacenter(int datacenterId)
      ```

      Binds this scheduler to a datacenter


    - #### setWorkflowEngineId

      ```
      public void setWorkflowEngineId(int workflowEngineId)
      ```

      Sets the workflow engine id

      Parameters:
      :   `workflowEngineId` - the workflow engine id


    - #### processEvent

      ```
      public void processEvent(SimEvent ev)
      ```

      Process an event

      **Overrides:**
      :   `processEvent` in class `DatacenterBroker`

      Parameters:
      :   `ev` - a simEvent obj


    - #### processVmCreate

      ```
      protected void processVmCreate(SimEvent ev)
      ```

      Process the ack received due to a request for VM creation.

      **Overrides:**
      :   `processVmCreate` in class `DatacenterBroker`

      Parameters:
      :   `ev` - a SimEvent object


    - #### processCloudletUpdate

      ```
      protected void processCloudletUpdate(SimEvent ev)
      ```

      Update a cloudlet (job)

      Parameters:
      :   `ev` - a simEvent object


    - #### processCloudletReturn

      ```
      protected void processCloudletReturn(SimEvent ev)
      ```

      Process a cloudlet (job) return event.

      **Overrides:**
      :   `processCloudletReturn` in class `DatacenterBroker`

      Parameters:
      :   `ev` - a SimEvent object


    - #### processCloudletCheck

      ```
      protected void processCloudletCheck(SimEvent ev)
      ```

      process cloudlet (job) check (not supported yet)

      Parameters:
      :   `ev` - a simEvent object


    - #### startEntity

      ```
      public void startEntity()
      ```

      Start this entity (WorkflowScheduler)

      **Overrides:**
      :   `startEntity` in class `DatacenterBroker`


    - #### shutdownEntity

      ```
      public void shutdownEntity()
      ```

      Terminate this entity (WorkflowScheduler)

      **Overrides:**
      :   `shutdownEntity` in class `DatacenterBroker`


    - #### submitCloudlets

      ```
      protected void submitCloudlets()
      ```

      Submit cloudlets (jobs) to the created VMs. Scheduling is here

      **Overrides:**
      :   `submitCloudlets` in class `DatacenterBroker`


    - #### processCloudletSubmit

      ```
      protected void processCloudletSubmit(SimEvent ev)
      ```

      Submits cloudlet (job) list

      Parameters:
      :   `ev` - a simEvent object


    - #### processResourceCharacteristicsRequest

      ```
      protected void processResourceCharacteristicsRequest(SimEvent ev)
      ```

      Process a request for the characteristics of a PowerDatacenter.

      **Overrides:**
      :   `processResourceCharacteristicsRequest` in class `DatacenterBroker`

      Parameters:
      :   `ev` - a SimEvent object


- Overview
- Package
- Class
- Use
- Tree
- Deprecated
- Index
- Help

- Prev Class
- Next Class

- Frames
- No Frames

- All Classes

- Summary:
- Nested |
- Field |
- Constr |
- Method

- Detail:
- Field |
- Constr |
- Method
